# The contribution of landscape features, climate and topography in shaping taxonomical and functional diversity of avian communities in a heterogeneous Alpine region

**DOI:** 10.1007/s00442-022-05134-7

**Published:** 2022-02-22

**Authors:** Matteo Anderle, Chiara Paniccia, Mattia Brambilla, Andreas Hilpold, Stefania Volani, Erich Tasser, Julia Seeber, Ulrike Tappeiner

**Affiliations:** 1 Institute for Alpine Environment, Eurac Research, Drususallee/Viale Druso 1, 39100 Bolzano/Bozen, Italy; 2 Department of Ecology, University of Innsbruck, Sternwartestrasse 15/Technikerstrasse 25, 6020 Innsbruck, Austria; 3 Dipartimento Di Scienze E Politiche Ambientali, Università Degli Studi Di Milano, via Celoria 26, 20133 Milano, Italy

**Keywords:** Agriculture intensification, Avian conservation, Habitat type, Landscape homogenisation, Land abandonment

## Abstract

Understanding the effects of landscape composition and configuration, climate, and topography on bird diversity is necessary to identify distribution drivers, potential impacts of land use changes, and future conservation strategies. We surveyed bird communities in a study area located in the Central Alps (Autonomous Province of South Tyrol, northeast Italy), by means of point counts and investigated taxonomic and functional diversity at two spatial scales along gradients of land use/land cover (LULC) intensity and elevation. We also explored how environmental variables influence bird traits and red-list categories. Models combining drivers of different types were highly supported, pointing towards synergetic effects of different types of environmental variables on bird communities. The model containing only LULC compositional variables was the most supported one among the single-group models: LULC composition plays a crucial role in shaping local biodiversity and hence bird communities, even across broad landscape gradients. Particularly relevant were wetlands, open habitats, agricultural mosaics made up of small habitat patches and settlements, ecotonal and structural elements in agricultural settings, and continuous forests. To conserve bird diversity in the Alps, planning and management practices promoting and maintaining small fields, structural elements, and a mosaic of different LULC types should be supported, while preserving continuous forests at the same time. Additionally, pastures, extensively used meadows, and wetlands are key to conservation. These strategies might mitigate the impacts of global change on bird diversity in the Alps and in other European mountain areas.

**Supplementary Information:**

The online version contains supplementary material available at 10.1007/s00442-022-05134-7.

## Introduction

Worldwide, biodiversity has been declining more rapidly in the last decades than at any time in recent human history (Barnosky et al. 2011), and anthropogenic land use/land cover (LULC) changes have had extreme impacts (Newbold et al. 2015). Habitat loss and fragmentation are critical drivers of biodiversity declines, and largely result from agricultural intensification in favourable areas, or rural abandonment of marginal ones (Tasser et al. 2007; Newbold et al. 2015). Both these opposing processes lead to landscape homogenisation (Brambilla 2019), resulting in severe losses of habitat diversity (Kujawa et al. 2020), and lower the beta diversity of animal communities (Burgess and Maron 2016)*.* Habitat fragmentation negatively influences abundance, movements, and persistence of many bird species (Villard et al. 1999) by disrupting connectivity between habitats (Amini Tehrani et al. 2020) and reduces native biodiversity and ecological integrity (Noss and Cooperrider 1994). Moreover, habitat loss and fragmentation also affect functional diversity (Flynn et al. 2009).

Understanding the impacts of habitat loss and fragmentation on biodiversity is therefore essential. However, several driving factors (e.g., topography, climate, landscape composition or configuration) contribute synergistically or antagonistically to biodiversity patterns. These drivers and their relative changes often act together, making it difficult to disentangle overlapping effects (de Chazal and Rounsevell 2009). Also, effect predictions may change at different spatial scales, both in terms of landscape attributes (Debinski et al. 2001) and climate (Brambilla et al. 2019). Only few studies evaluated the combined effect of factors (Barras et al. 2021; Ceresa et al. 2021), whereas most studies focused on single factors (Cabral et al. 2021) or single habitats (Jacoboski and Hartz 2020). Specifically, LULC and climate change are mostly investigated separately, resulting in a high risk of over- or underestimating their relative influence (de Chazal and Rounsevell 2009). Thus, detailed information on LULC effects on species abundance and functionality, simultaneously considering climate and topography, is required to accurately predict the effects of LULC changes and disentangle their importance for biodiversity from other drivers (Brambilla et al. 2020a). This combined approach is also key to effective conservation and management (Doley 2010), as is the evaluation of landscape composition or configuration effects over environmental gradients (Rüdisser et al. 2012). This is particularly relevant in mountain landscapes, where elevation gradients shape complex environments (Liu et al. 2018), even though this has rarely been explored (Amini Tehrani et al. 2020). In the European Alps, a biodiversity hotspot hosting several endemic species (Jenkins et al. 2013), LULC changes are occurring along broad elevation gradients, and across very different environments (Pecher et al. 2013).

In this study, we assess the effects and the importance of landscape composition and configuration, climatical and topographical drivers, on bird functional and taxonomic diversity across different landscapes and elevation gradients in the Alps. Birds are a diverse, well known and easily censused group, that are excellent indicators for global biodiversity trends (BirdlifeInternational 2020). Birds play an essential role in key ecological processes, including seed dispersal, pollination, pest control, nutrient cycling, and scavenging (S̜ekercioğlu et al. 2016). They are highly sensitive to environmental changes, and respond quickly to habitat alteration, including climate and LULC changes (Regan et al. 2015; S̜ekercioğlu et al. 2016; Scridel et al. 2018). To investigate the effects of environmental drivers on biological communities by combining functional and taxonomic diversity metrics offers a broader understanding of such effects and of potential consequences for ecosystem functions (Siriwardena et al. 2019; Jacoboski and Hartz 2020).

In this study, we aim to investigate how LULC, climatical, and topographical drivers shape bird diversity in European mountain landscapes. Specifically, we want to understand (1) which of these factors exert the strongest effects, and (2) how they impact bird diversity, as well as to address (3) how those drivers select for specific traits and are related to different threat level according to bird red-list categories. Understanding the impact of LULC patterns on biological community structure, taxonomic and functional diversity, is key to the effective management of changing ecosystems (Lee and Martin 2017)*,* and is particularly urgent for a biodiversity hotspot undergoing important transformations such as the Alps (Payne et al. 2017)*.*

## Materials and methods

### Study area and study sites

**Fig. 1:**
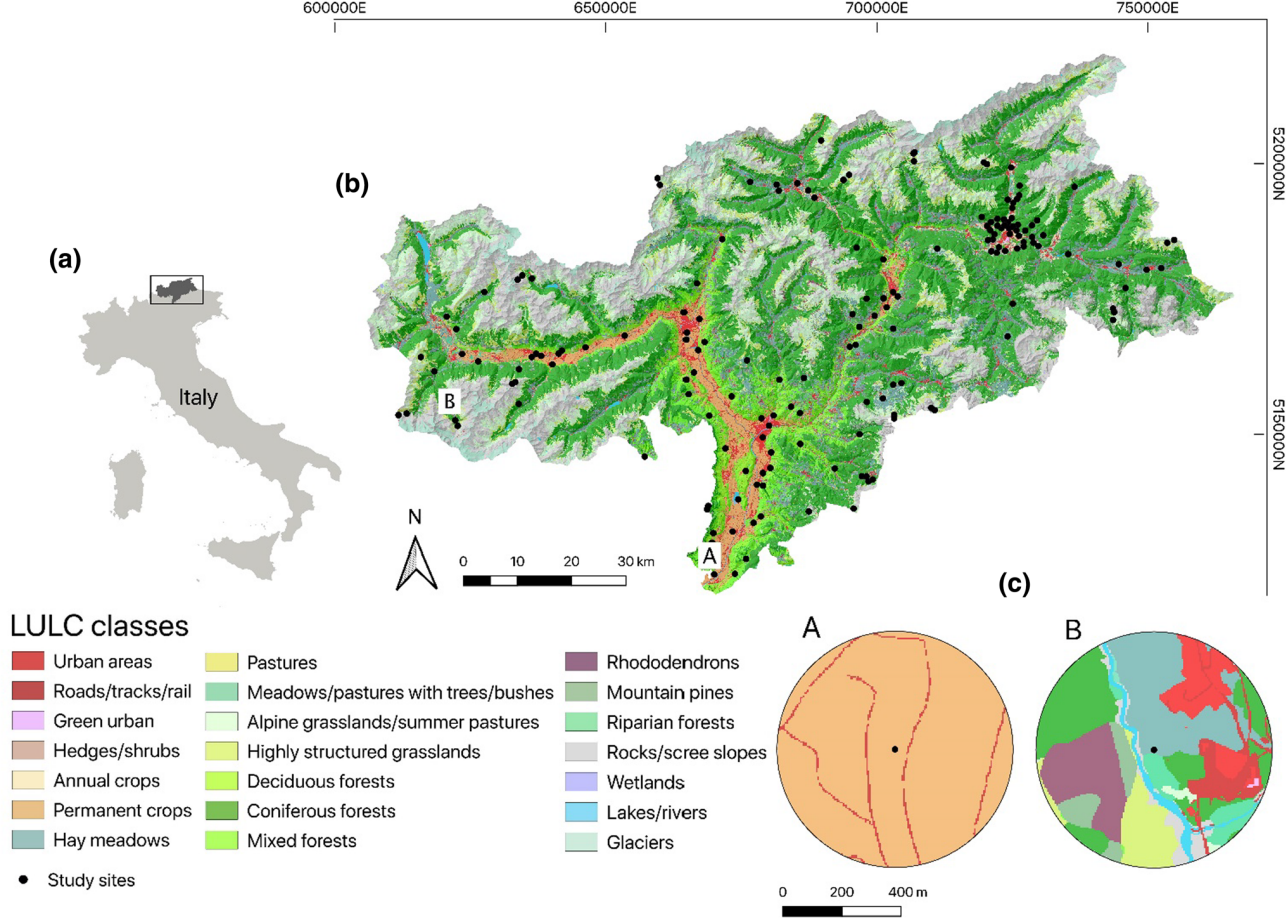
**a** Autonomous Province of South Tyrol in Italy, **b** the 168 study sites, **c** two landscape examples (A and B) showing the spatial details of land use/land cover (LULC) mapping

The study was carried out in the Autonomous Province of South Tyrol (Italy), in the Central Alps (Fig. 1). It extends over approx. 7400 km^2^, with a broad elevation range (194–3905 m a.s.l.). Landscapes comprise forests (42.7%), natural and seminatural lands such as alpine grasslands, rocks, freshwater habitats and glaciers (overall 39.6%), intensive agricultural land (13.4%), while settlements are rather limited (4.3%). Most agricultural areas are meadows (64.3%), followed by orchards (19.1%), pastures (6.3%), vineyards (5.6%), and annual crops (3.9%). Crops are mainly located in valley bottoms, whereas meadows and pastures are found from mountainsides to the subalpine and alpine belts. Data were collected in the framework of the project *Biodiversity Monitoring South Tyrol* (BMS 2021). According to the main habitats 168 study sites were selected by a stratified and random site-selection approach (see BMS 2021 and Fig. 1).

### Bird survey

We surveyed bird communities by means of 10-min point-counts (Bibby 2000; Sutherland 2006), considering all birds within a 100 m-radius from the site (Fornasari and Mingozzi 1999). To avoid double counting of the same individuals, the minimum distance between neighbouring sites was set at 800 m. Surveys were carried out between mid-April and mid-July in 2019–2020, depending on local conditions. Every year we sampled the same number of sites per habitat type (see BMS, 2021). We started earlier in valley-bottom sites and later in the Alpine sites, as bird breeding season starts later with increasing elevation (Assandri et al. 2017a; Ceresa et al. 2020b). Counts started shortly after sunrise (5.30 a.m.), and were completed before 11 a.m. For sites at lower elevations we performed three visits, but only two for upland sites (subalpine or alpine) because of the much shorter breeding season at higher elevation. Between subsequent visits, at least 15 days passed, and the order of site-surveys was changed. Adverse weather conditions (moderate/strong wind or heavy rain/snow) were avoided.

### Bird traits

Species-trait approaches focus on functional aspects of biodiversity, and constitute an additional tool to the traditional taxonomic approach (Siriwardena et al. 2019; Jacoboski and Hartz 2020). Following previous studies (Jacoboski and Hartz 2020; Altamirano et al. 2020), we selected traits that can affect species’ ability to respond to habitat changes (e.g., landscape homogenisation may select for species with smaller size, a generalist diet, shorter generation time, and higher dispersal abilities). The traits considered were diet, foraging substrate, mean body mass, broods per year, nest type, habitat use during breeding season, territoriality, migration strategy, and specialization. We also included the red-list categories of birds, using threat categories from the Red List of 2020 for birds breeding in South Tyrol (Ceresa and Kranebitter 2020). See Table S1 for sources and full descriptions of traits.

### Bird diversity indices

We removed records related to species observed only flying over sites or occurring exclusively as migrants (Table S2). A functional dispersion index (Fdis, Laliberté and Legendre 2010) was calculated using the avian traits, based on foraging behaviour, morphology, ethology, and breeding behaviour (Kim et al. 2020). We used Fdis, calculated using the ‘*FD*’ package in R (Laliberté et al. 2014), to describe the overall functional diversity in the community, since it measures the mean distance of species in a community to the centroid of all species in that community, being less affected by extreme values than other functional indices. Bird species richness (Sric) was calculated from the total number of species observed at each site during all visits. For calculating the Shannon diversity index (Shan) we chose the observation with the highest number of individuals per species out of all visits at a given site to avoid double count of the same individual during multiple visits. Sric and Shan were calculated using the ‘*Vegan*’ package in R (Oksanen et al. 2020).

### Environmental variables

We prepared a LULC map by integrating different sources (Table 1 and Table S5). The minimum mapping unit was 25 m^2^, and we differentiated the LULC into 21 classes potentially important for bird ecology in the Alpine region (Assandri et al. 2017b; Brambilla et al. 2020c). We considered three variable types (topo-climatical, compositional, and configurational) evaluated at two spatial scales, considering radii of 100 m (3.14 ha) and 400 m (50.24 ha) around each site. The small radius reflects the territory size of many passerine species, which often defend territories of a few hectares during the breeding season (e.g., Bocca et al. 2007; Brambilla and Ficetola 2012; Pestka et al. 2018), while the larger one reflects the wider home ranges of larger birds (e.g., Wiktander et al. 2001; Bocca et al. 2007). Furthermore, the effects of some factors may be scale-dependent (e.g., microclimate conditions or spatial configuration).

**Table 1:** Environmental variables descriptions, units, and whether they were tested in the model approach or discarded for high correlations. For references see Table S5

Type	Variable	Name	Included	Description	Unit
Topo-climatical	SolarRad	Potential solar radiation	Yes	Sum of direct, diffuse, and reflected radiation due to sun irradiance, according to incidence solar angle, and the shadowing effect of topography. It was computed for a reference day (21st June) using the command r.sun in GRASS GIS (GRASS Development Team 2020)	Wm^−2^
	TMAMme	Mean spring temperature	No	Mean temperature March-June (mean of daily temperature)	°C
	TANNUALme	Mean annual temperature	No	Mean temperature during the year (mean of daily temperature)	
	Elev	Elevation	No	Elevation extracted by site using QGIS (QGIS Development Team 2020)	m a.s.l.
	Slope	Slope	Yes	Mean slope within 100 and 400 m buffer using QGIS (QGIS Development Team 2020)	°
	AnnPrec	Mean annual precipitation sum	Yes	Interpolated values of mean annual precipitation sum (basis data: 1981–2010)	mm a^−1^
Compositional	Glacier	Glaciers	No	Percentage of LULC classes within the buffer	%
	Urb	Urban areas	Yes		
	GreenUrban	Green urban	No		
	AlpGrass	Alpine grasslands and summer pastures	Yes		
	AlpShr	Highly structured grasslands	Yes		
	HedgShru	Hedges and/or shrubs	Yes		
	Meadow	Hay meadows	Yes		
	Pasture	Pastures	Yes		
	MeadPastTree	Meadows and pastures with trees and/or bushes	No		
	AnnCult	Annual crops	Yes		
	PermCult	Permanent crops	Yes		
	RocScr	Rock/ screen slopes	Yes		
	DecFor	Deciduous forests	Yes		
	ConFor	Coniferous forests	Yes		
	RipFor	Riparian forests	Yes		
	MixFor	Mixed forests	No		
	MountPine	Mountain pines	No		
	Rhod	Rhododendrons	No		
	Wet	Wetlands	Yes		
	LakRiv	Lakes and rivers	Yes		
	Roads	Roads, tracks and rail	Yes		
Configurational	ED	Edge density	Yes	Sum of the edges of all LULC classes divided by the area of the buffer. It includes buffer boundary segments representing 'true' edge only (i.e., abutting patches of different classes)	m ha^−1^
	AREA_MN	Mean patch area	Yes	Buffer area divided by the total number of patches inside	ha
	PR	Patch richness	Yes	Number of different LULC? types present within the buffer boundary	n
	SHDI	Shannon diversity index	Yes	$SHDI=-\sum_{i=1}^{m}({P}_{i}*\mathrm{ln}{P}_{i})$ ${P}_{i}$= proportion of the area occupied by LULC type (class)$i$	index
	SHEI	Shannon evenness index	No	$SHEI=\frac{-\sum_{i=1}^{m}\left({P}_{i}*\mathrm{ln}{P}_{i}\right)}{\mathrm{ln}m}$ ${P}_{i}$= proportion of the area occupied by LULC type (class) $i$ $m$ = number of LULC types (classes) present in the area, excluding the buffer border if present	

Following other studies (Veach et al. 2017; Amini Tehrani et al. 2020) topo-climatical variables included: elevation, slope, potential solar radiation, mean annual precipitation, mean spring precipitation, and mean annual temperature. Landscape composition variables were estimated from the LULC map as the proportional cover of each LULC class. Following Fahrig et al. (2011), we selected Shannon diversity and Shannon evenness, the mean patch area, edge density and patch richness as configurational variables. Compositional and configurational variables were calculated using Fragstats 4.2 software (McGarigal 2015). See Table 1 for variable descriptions.

### Data analysis

Following Zuur et al. (2010) we standardised all independent variables to better evaluate collinearity and relative responses (Cade 2015). We removed all variables with high collinearity (Spearman's Rho ≥ 0.68, Dormann et al. 2013) from the database and at each modelling step we evaluated multicollinearity according to variance inflation factors (VIFs), and discarded the most problematic ones (VIF > 4; Zuur et al. 2009, see Appendix S1). We built an accumulation curve using the *‘iNEXT’* package (Hsieh et al. 2016) to judge the sampling adequacy of our bird surveys for all sites (Fig. S7) as well as for the alpine and subalpine sites (Fig. S8).

We used a two-step approach, that allowed us to compare the support for different types of variables, and to find the most important variables within each type, while reducing the number of predictors at each step. Initially, we separately tested the effect of different types of variables on the three dependent variables Fdis, Shan, and Sric, at two spatial scales, using Linear Models (LM) for Fdis and Shan, and Generalised Linear Models (GLM) with a Poisson distribution for Sric (since these are count data; Zuur et al. 2013). At each step, we built all possible models with the dredge function in the R package ‘*MuMIn*’ (Barton 2020).

**Fig. 2:**
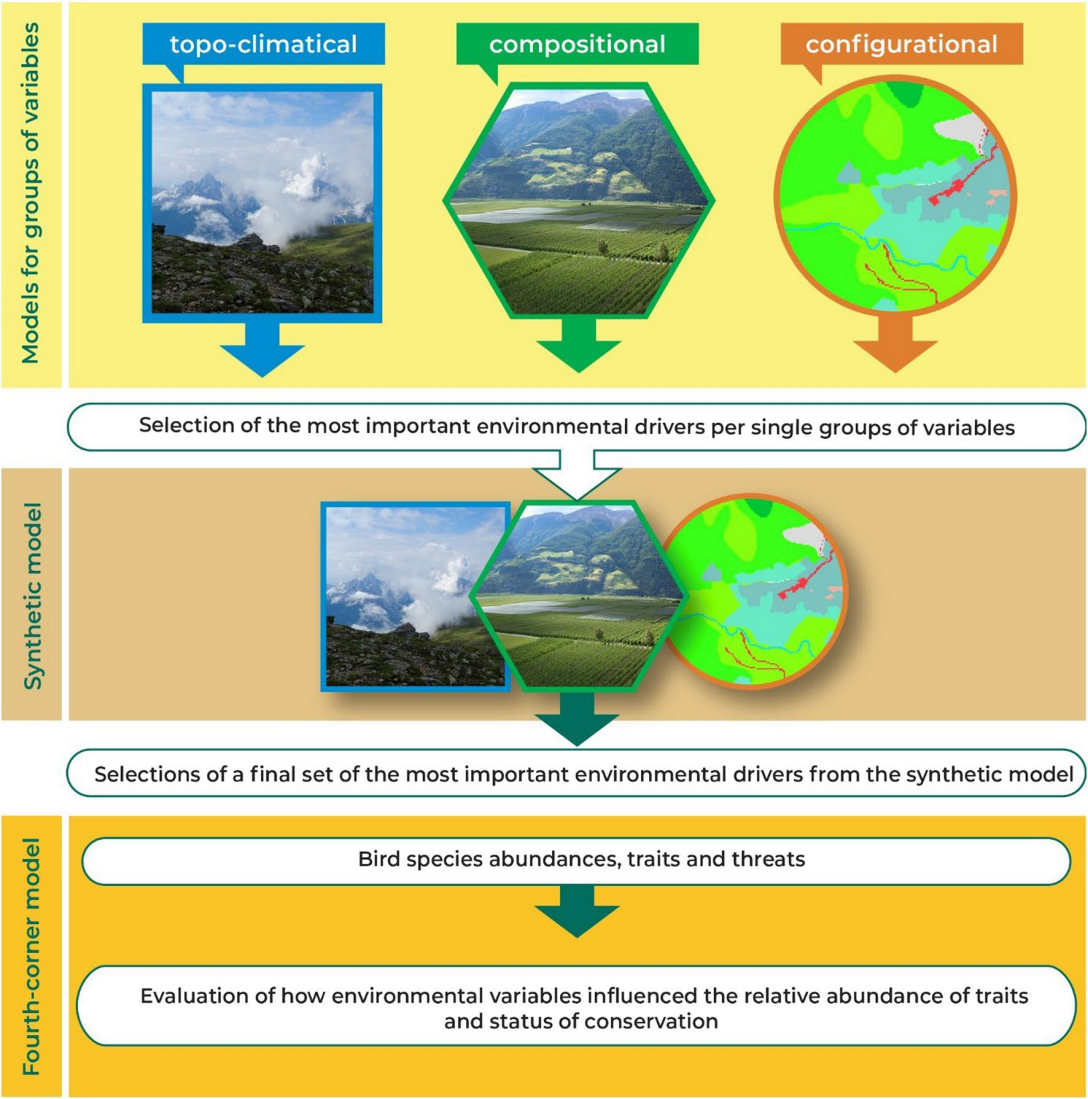
Scheme showing the statistical framework adopted to evaluate the effects of different types of environmental variables (topo-climatical, LULC compositional, and configurational) on the three bird diversity indices (species richness, Shannon diversity and functional diversity), and on bird traits and red-list categories. This approach was used at two different spatial scales (100 and 400-m radii)

An information-theoretic approach was adopted to perform a model selection based on the Akaike's information criterion (Burnham and Anderson 2004), corrected for small sample size (AICc). As a first step, we selected the most supported models (ΔAICc < 2) within each type of predictors, after excluding uninformative parameters, i.e., those whose inclusion in the model resulted in an increase in the ΔAICc value, even if the increase in AICc was lower than 2 (Arnold 2010). We carried out model averaging among the remaining models (all models with ΔAICc < 2), or took the remaining unique, most supported model, if no other models showed similar support (ΔAICc < 2). In the second step, we selected from each group all the environmental variables included in the averaged or most supported model and re-ran the same procedure. We defined these models combining the most important predictors from all the different types as “synthetic models” (Assandri et al. 2017a; Brambilla et al. 2020a). We thus obtained six synthetic models (one for each dependent variable, at both scales), by averaging (full average) the most supported ones (ΔAICc < 2), or by taking the most supported if there were no alternative models with similar support (Fig. 2).

Finally, using the ‘*traitglm*’ function in ‘*mvabund*’ (Wang et al. 2012) we evaluated how the environmental variables included in the synthetic models influenced the presence of traits and red-list categories of bird species by adopting a model-based approach to the fourth‐corner analysis (Brown et al. 2014). The fourth-corner model relates species traits and landscapes attributes by fitting a predictive model of species abundance as a function of matrices of environmental variables, species traits, and their interaction (‘*mvabund*’ R package; Wang et al., 2012). This method uses an extension of a GLM, fitting a single predictive model to all species across all sites simultaneously. Three matrices of environmental data, species abundance data, and species trait data were used to calculate a fourth matrix of trait–environment interaction coefficients, or fourth-corner terms (Wang et al. 2012). For visual interpretation we generated two heat-maps (one applied to traits and one to red-list categories), and used the LASSO penalty to remove interactions that failed to improve model fit (Wang et al. 2012). To test the statistical significance of the overall relationship between variables and trait or red-list category, we computed a Monte-Carlo randomisation test with 999 permutations (Wang et al. 2012). Lastly, we checked for potential patterns of spatial autocorrelation in model’s residuals by means of a variogram (Dormann et al. 2007). Residuals showed no pattern at all, suggesting the lack of spatial autocorrelation. All the analyses were done with R version 3.4.1 (R Development Core Team 2019).

## Results

The final dataset included 4,494 individuals belonging to 110 species. The Chaffinch (*Fringilla coelebs*) was the most observed species with a total of 330 individuals; some of the rarest were Corn crake (*Crex crex*), Little bittern (*Ixobrychus minutus*), and Eurasian three-toed woodpecker (*Picoides tridactylus*) which were recorded only once. At the species richest study site (a wetland), we counted 27 species, and at the poorest ones 3 species (three alpine sites). The accumulation curves indicated that the sampling was adequate and complete (Fig. S7 and Fig. S8).

### Modelling the effect of different drivers on avian communities

At both spatial scales, and for all the dependent variables, the synthetic model had the overall lowest AICc (Table 2). Comparing the models containing only a single type of variables, again at both spatial scales and for all dependent variables, the models based on landscape compositional variables exhibited the lowest AICc. At the smaller scale the topo-climatical model, and at the larger scale the landscape configuration model were the most supported after the landscape compositional model.

**Table 2:** AICc and *R*^2^ of synthetic models, and of models including only single type of environmental variables

Scale	Dependent variable	Type of model	AICc	R^2^
100 m radius	Sric	Topo-climatical	973.90	
		Compositional	917.08	
		Configurational	994.42	
		Synthetic	911.03	0.54
	Shan	Topo-climatical	204.86	
		Compositional	162.33	
		Configurational	216.45	
		Synthetic	150.96	0.46
	Fdis	Topo-climatical	− 582.88	
		Compositional	− 650.15	
		Configurational	− 578.9	
		Synthetic	− 651.04	0.47
400 m radius	Sric	Topo-climatical	973.90	
		Compositional	887.82	
		Configurational	949.11	
		Synthetic	875	0.63
	Shan	Topo-climatical	204.86	
		Compositional	141.05	
		Configurational	191.73	
		Synthetic	129.10	0.52
	Fdis	Topo-climatical	− 582.88	
		Compositional	− 647.91	
		Configurational	− 595.10	
		Synthetic	− 653.06	0.45

Data were grouped firstly for spatial scales, and secondly for dependent variables (Sric = species richness, Shan = Shannon diversity index, and Fdis = functional diversity index). For more details on all the most supported models (AICc < 2) among all the possible ones see Table S3

According to the synthetic models, topo-climatical variables did not show consistent effects, apart from a negative impact of mean annual precipitation on all indices at the small scale (but note a positive effect on species richness at 400 m).

For the compositional variables, Alpine grassland and summer pastures had positive effects on Fdis and negative ones on Shan and on Sric. Highly structured grasslands, hay meadows, and pastures always showed positive influences on Fdis and Sric. On the contrary, permanent crops exerted negative effects on all indices, while annual crops had positive effects on Fdis. Deciduous forests positively impacted Fdis, while hedges and shrubs, coniferous and riparian forests had negative effects. Lakes/rivers and wetlands had positive effects on all indices. Roads/tracks/rail positively affected Fdis and Shan, whereas urban areas had positive effect on Fids and negative on Shan.

Configurational variables included in the synthetic models were patch richness (with positive effects on Shan and Sric), and patch area (negative effects on Fdis and Shan). For a more complete interpretation see Table 3 and Table S4.

**Table 3:** Graphical representation of the responses of dependent variables to predictors in the synthetic models. “ + ” and “-” represent positive and negative effects, respectively (see Table 1 and S4, and Fig. S1-S6, for details)

Type of variables	Environmental variables	Functional diversity		Shannon diversity		Species richness	
		100-m	400-m	100-m	400-m	100-m	400-m
Topo-climatical	Potential solar radiation	− 0.01					
	Mean annual precipitation sum	− 0.01		− 0.13		− 0.02	+ 0.05
Compositional	Alpine grasslands and summer pastures	+ 0.01		− 0.13	− 0.19	− 0.13	− 0.16
	Rock/screen slopes	− 0.002		− 0.17	− 0.16	− 0.20	− 0.17
	Highly structured grasslands	+ 0.003	+ 0.003				
	Hay meadows	+ 0.02	+ 0.02				+ 0.02
	Pastures						+ 0.01
	Annual crops	+ 0.01	+ 0.002	− 0.10	− 0.08		− 0.03
	Permanent crops	− 0.001		− 0.12	− 0.05	− 0.06	
	Deciduous forests	+ 0.0006					
	Coniferous forests	− 0.0005					
	Riparian forests		− 0.004				
	Hedges and/or shrubs	− 0.003	− 0.003				
	Lakes and rivers	+ 0.007	+ 0.01		+ 0.07	+ 0.01	+ 0.07
	Wetlands	+ 0.008				+ 0.05	
	Roads, tracks and rail	+ 0.004		+ 0.02			
	Urban areas	+ 0.02	+ 0.02	− 0.12	− 0.10		
Configurational	Patch richness			+ 0.05	+ 0.10	+ 0.07	+ 0.12
	Mean patch area	− 0.002	− 0.009		− 0.03		

### Modelling environmental drivers on bird red-list categories

**Fig. 3:**
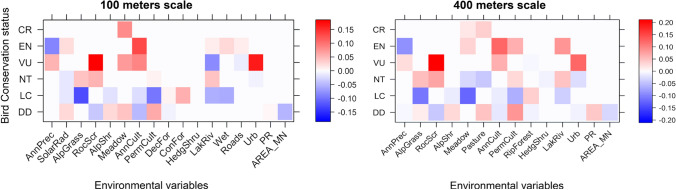
Relationships of red-list categories and environmental variables. Darker colours represent stronger associations (blue negative correlations, red positive ones). For red-list categories see the text; for variables see Table 1

In the fourth-corner analysis (Fig. 3) mean annual precipitation showed negative and positive associations, respectively, with endangered (EN) and vulnerable (VU) species at both scales. Open areas were associated with bird communities with many threatened species: hay meadows were positively related with critical endangered (CR) at both scales, with EN and VU respectively at large and small scales, and pastures with CR species at largest scale. Additionally, Alpine grasslands/summer pastures at both scales, and hay meadows at 400 m, were negatively associated with least concern (LC) species. Annual crops were positively associated with EN and VU at both scales, negatively with LC at 400 m, and moderately negatively at 100 m. Lakes/rivers were associated with EN and near threatened (NT) species in at largest scale; wetlands with EN at small scale. Configurational variables did not show association with red-list categories.

### Modelling environmental drivers on bird traits

**Fig. 4:**
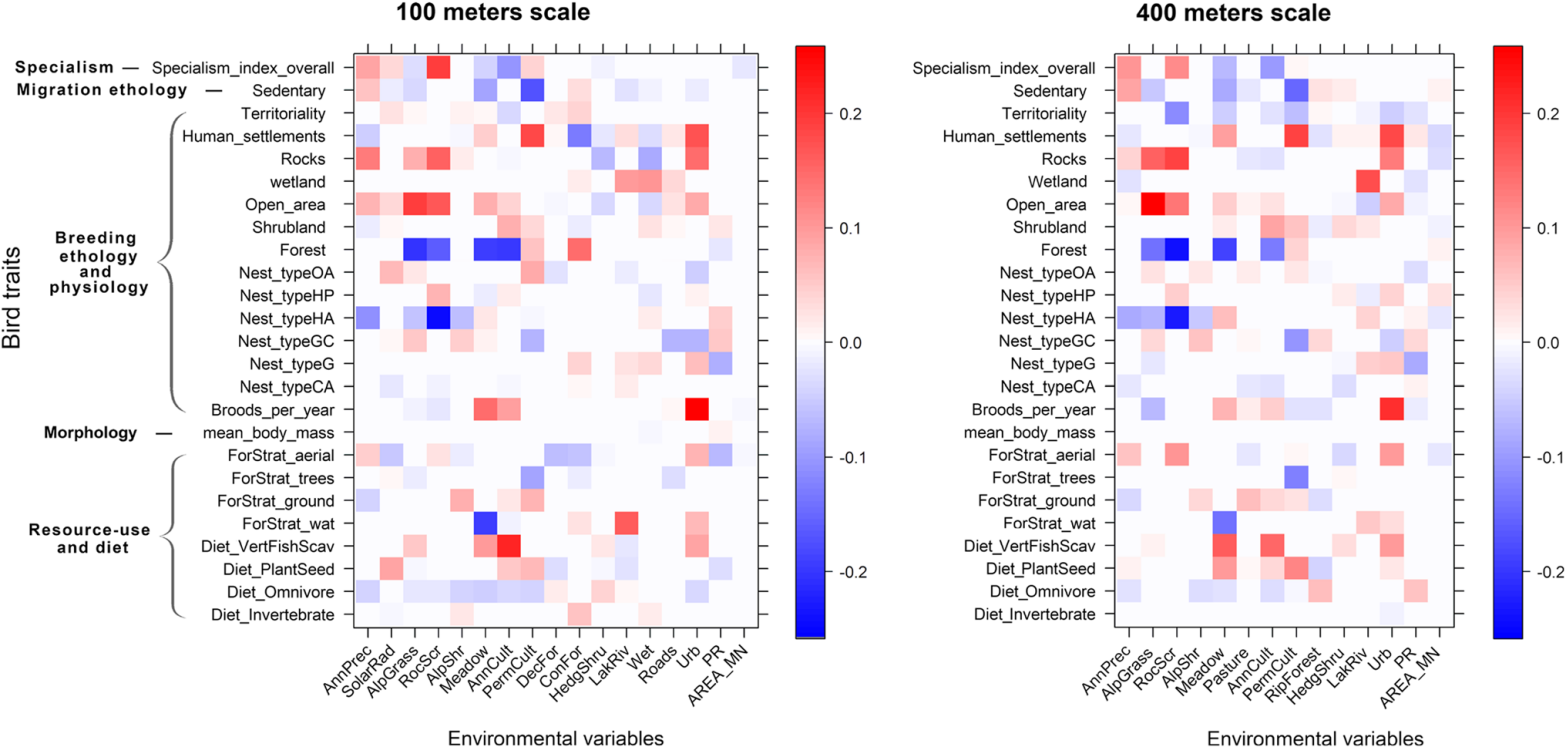
Relationships between bird traits and environmental variables derived from the synthetic models. Darker colours represent stronger associations. Blue colour represents negative correlations; red colour represents positive ones. For bird traits see Table S1; for variables see Table 1

Environmental parameters associated with bird traits revealed rather unexpected results (Fig. 4). Annual crops were associated with low specialization at both scales. Non-sedentary species were abundant in permanent crops at both scales. Open area species were more strongly associated with Alpine grasslands/summer pastures at both scales. Shrubland specialist species occurred at both scales in annual crops. Forest species were positively associated with permanent crops at both scales, and with less patches at 100 m, and larger mean patch area at 400 m. A strong positive correlation emerged between permanent crops and birds mainly nesting in open woodlands, in bushes and trees, while species that nest on or close to the ground were fewer in permanent crops, and moderately common in open habitats. Considering diets, at both scales, birds that feed in the tree layer were negatively affected by permanent crops. Birds feeding on the ground were associated with annual and permanent crops, and with highly structured grasslands. Birds with a diet dominated by plants and seeds were associated with both annual and permanent crops.

## Discussion

Investigating how drivers such as land use/land cover (LULC) composition and configuration, topography, and climate affect biological communities is essential to decipher which factors exert the strongest effect on biodiversity at different spatial scales. Here, we focused on bird communities considering three diversity indices (species richness, Shannon diversity, and functional diversity) across broad landscape gradients determined by elevation, topography, and local climate. Especially the functionality index allowed us to assess the diversity of the bird community beyond the simple species diversity at a site. The fourth-corner analysis evaluated the link between environmental drivers and threatened species and/or species with unique traits. Addressing the distribution of traits across different gradients to identify priority conservation action provides a more comprehensive assessment of the quality of an ecosystem and of the resilience of a bird community than species richness alone (Veach et al. 2017).

For all three diversity indices at both spatial scales, the synthetic model was the most supported one, denoting the concurrent effect of different environmental drivers. This is consistent with the importance of such predictors, and of the relative interactions, reported from studies focusing on less heterogeneous environments (Jongsomjit et al. 2013; Mantyka-Pringle et al. 2015), and in particular for Alpine bird communities (Chamberlain et al. 2016; Scridel et al. 2018). The LULC compositional model was invariably the most supported among the single-group models. LULC composition plays a crucial role in shaping local biodiversity and hence bird communities (Santana et al. 2017), even across such a broad environmental gradient. Topo-climatic models were also important, especially at the smaller scale, where the effect of topography and climate are likely to affect mesoclimatic conditions, while landscape configurational models were more important at the larger scale. These findings are especially relevant for many mountain systems, where land abandonment, management intensification, habitat fragmentation, as well as climate change, are posing severe threats to biodiversity (Chamberlain et al. 2016; Scridel et al. 2018) and are predicted to be the most impacting pressures also for the decades to come (Newbold 2018).

Topo-climatical drivers in the synthetic models mostly acted at the small scale. The only exception was mean annual precipitation, which positively affected bird species richness at the 400 m scale, while negatively impacting all three indices at the 100 m scale. The interaction between precipitation and topographical traits likely led to relevant variations of local climates. Climate predictors were highly intercorrelated; we retained precipitation in the models because in the Alps mean annual precipitation and continentality vary greatly with geographical location, with different patterns of variation along elevation gradients. Inner valleys are characterised by very continental climates, whereas other regions in the Alps, especially the peripheral ones, exhibit more oceanic climates, with much higher precipitation levels. This gradient is very pronounced also within our study area, with yearly precipitation ranging from c. 500 mm per year (Vinschgau) to > 1600 mm per year (Ulten Valley; Rubel et al. 2017). Furthermore, the fourth-corner analysis revealed a pattern of opposite association between endangered and vulnerable species with mean annual precipitation; in our study endangered species were found mainly at lower elevation and generally in drier valleys such as *Dryobates minor* and *Sylvia communis*, while vulnerable species were found generally in higher and wetter areas, such as *Montifringilla nivalis* and *Cettia cetti*.

LULC composition variables were abundant in the synthetic models, denoting their importance, particularly along gradients of very heterogeneous landscapes. The recent regional Red List of breeding birds reported for the study area that almost half of the bird species listed suffer from habitat destruction due to LULC change, disappearance of uncultivated areas, riparian vegetation and hedges, while another large portion of species suffers from intensive management of farmed areas. These findings were corroborated by our results. Alpine grasslands and summer pastures showed a positive relationship with functional diversity, probably because a relatively high number of specialist species, with very narrow ecological niches occur in these habitats, resulting in high functional diversity (Altamirano et al. 2020). Moreover, highly structured grasslands, hay meadows and pastures always showed positive effects on all diversity indices. This result demonstrated the overall importance of open areas, and of their management, for both functional and taxonomic bird diversity (Assandri et al. 2019). At both spatial scales, open-area habitats were most closely associated with threatened species, and negatively with non-threatened ones. The same is true for water-dominated habitats, which are rich in threatened species (Brusa et al. 2019; Morganti et al. 2019). Our synthetic models suggested that fresh-water habitats had strong positive influences on all dependent variables. Wetlands, lakes, and rivers are important hotspots of biodiversity in the Alpine region, representing key sites for bird conservation (Brusa et al. 2019). Therefore, preserving open areas and wetlands would not only increase species number/diversity and functional diversity in bird communities, but would also benefit a large proportion of threatened species. Annual crops were positively correlated with functional diversity and negatively with the other indices, while permanent crops had negative effects on all diversity indices. In the Alpine region, annual crop fields are mostly small-sized and often part of environmental mosaics, with natural or seminatural elements, showing positive effects on biodiversity (Ceresa et al. 2012). These mosaics offer more niches, increasing the functionality of the bird community. On the contrary, permanent crops are the most intensive agriculture in the region, being associated with a severe landscape homogenisation (Tasser et al. 2009), and thus harbour poorer bird communities. Annual crops were also positively associated with vulnerable and endangered bird species, consistently with the local status of farmland birds (Ceresa and Kranebitter 2020; Ceresa, et al. 2020a). In annual and permanent crops, birds mostly forage on the ground, feeding on plants and seeds, and mainly nest on open-arboreal areas, in bushes and trees. Heterogeneous agricultural landscapes, with more structural elements, and different LULC patches, are key to conserve avian communities in these landscapes. Coniferous forests negatively and deciduous forests positively affected functional diversity. Broadleaved forests are more heterogeneous and variable in the Alpine region, offering more and diverse niches for breeding birds (Winkler 2005). Our data suggested that forest specialists may avoid fragmented and discontinuous forests, preferring continuous forest areas, as more strongly evident in other studies about forest specialists (Bełcik et al. 2020). Hedges and shrubs, as well as riparian forests, had negative effects on functional diversity and hardly showed any critical effects on red-list categories and bird traits. In the study area, both habitats are predominantly found in depauperated landscapes, such as valley bottoms close to intensively managed agricultural areas. The negative effect associated with hedgerows is indeed likely due to their predominant occurrence in simplified agricultural landscapes of the study area; their occurrence may hence indicate an intensification context, not entirely captured by other variables. We do not believe that such a negative association is related to a true negative effect exerted by hedgerows. Riparian forests often suffer inadequate management, resulting in a very low degree of naturalness, and in the study area are often highly fragmented or highly urbanised, offering few ecological niches (Larsen 2017). Urban areas, mostly represented by small settlements or even single buildings, offer more niches and were positively related to functional diversity. Villages in agricultural areas or in heterogeneous landscapes increase landscape complexity (Tasser et al. 2009). Roads show a positive correlation with both functional diversity and taxonomic diversity (Shannon). This could be partially due to an increase in bird detectability along roads. However, roads, tracks, and rails often have an ecotonal effect on the surrounding landscapes (Dániel-Ferreira et al. 2020) and are lined with narrow strips of embankments or shrubbery, providing potential habitats for birds (Coffin 2007).

Landscape configurational variables largely entered in our synthetic models at both scales, confirming the importance of such factors even along broad environmental gradients. This is a particularly important result, as assessments usually evaluate the potential importance of landscape configuration over homogeneous contexts, or at a single spatial scale. We found a strong and positive effect of the number of patches of different LULCs on Shannon diversity and bird species richness. Expectedly, a higher landscape heterogeneity resulted in a higher species diversity (Redlich et al. 2018). The mean patch area exerted a strongly negative effect on bird functional diversity: the more configurationally heterogeneous an area, the higher the number of species and functional groups that could be expected (Devictor and Jiguet 2007). This pattern is consistent with the mosaic concept (Duelli 1997), which theorises that highly heterogeneous landscapes can harbour a more diverse and specialised flora and fauna, and thus contribute to the overall diversity. This result shows that landscape heterogeneity is of great importance for bird communities in diverse areas such as the Alps.

## Conclusions

For conservations strategies, we strongly recommend that landscapes surrounding agricultural and anthropogenic landscapes should be managed to maintain or (re)generate heterogeneous mosaics, with smaller and diversified patch sizes (avoiding larger continuous patches of single LULC). In that sense, some measures implemented in Rural Development Programmes of different Alpine regions, focusing on crop diversification and on the restoration of natural or seminatural habitats, such as ponds, shrub patches, grasslands, could provide an important contribution to this objective. On the other hand, in forest habitats efforts should be made to minimise habitat fragmentation by keeping woodlands as continuous as possible. The opposite association pattern between precipitation and endangered and vulnerable species, respectively, suggests that threatened species are associated with different climates, and hence conservation efforts need to encompass the broad climatic gradient. Strategies aimed at promoting birds and biodiversity in the Alpine region, undergoing dramatic changes, should acknowledge three key points. First, the importance of landscape heterogeneity in agricultural and anthropogenic areas should be promoted, giving priority to patches of open habitats and structural elements. In this sense, agricultural policies such as Rural Development Programmes could be crucial (Concepción et al. 2020; Rotchés-Ribalta et al. 2021). They should ideally consider the complementary effects of landscapes heterogeneity and the importance of small patch mosaics for the taxonomic and functional diversity of farmland birds. Second, the key role of wetlands must be recognized; their conservation and, where needed, restoration and proper management should be part of landscape and conservation strategies (see Morganti et al. 2019; Brambilla et al. 2020b, c for similar areas). Third, in the case of forest, habitat continuity and homogeneity are needed to support specialist bird species, and should be pursued by proper management and planning decisions.

## Supplementary Information

Below is the link to the electronic supplementary material.

Supplementary file1 (PDF 1220 KB)

## Data Availability

The datasets generated and analysed during the current study are available from the corresponding author on reasonable request.
