# Supplementary material for: The contribution of landscape features, climate and topography in shaping taxonomical and functional diversity of avian communities in a heterogeneous Alpine region
Source: Oecologia. 2022 Feb 22;199(3):499–512. doi: 10.1007/s00442-022-05134-7 (PMC9309150; doi:10.1007/s00442-022-05134-7)
Supplement: Supplementary file 1 — Supplementary file1 (PDF 1220 KB) [file 442_2022_5134_MOESM1_ESM.pdf]

Supplementary materials

The contribution of landscape features, climate and topography in shaping taxonomical and functional diversity of avian communities in a heterogeneous Alpine region

Matteo Anderle[1,2*], Chiara Paniccia[1], Mattia Brambilla[3], Andreas Hilpold[1], Stefania Volani[1], Erich Tasser[1], Julia Seeber[1,2] & Ulrike Tappeiner[1,2].

Affiliations:

1 Institute for Alpine Environment, Eurac Research, Drususallee/Viale Druso 1, I-39100 Bolzano/Bozen, Italy

2 Department of Ecology, University of Innsbruck, Sternwartestrasse 15/Technikerstrasse 25, A-6020 Innsbruck, Austria

3 Dipartimento di Scienze e Politiche Ambientali, Università degli Studi di Milano, via Celoria 26, I-20133, Milano, Italy

***Corresponding author**: Matteo Anderle, matteo.anderle@eurac.edu

Appendix S1

Independent variables correlation

For topo-climatical variables we removed TANNUALme, TMAMme and Elev from subsequent analyses due to high collinearity with AnnPrec (Spearman's Rho: TANNUALme = -0.78; $p < 0.001$; TMAMme = -0.78; $p < 0.001$; elev = 0.79; $p < 0.001$). Temperature variables (TANNUALme and TMAMme) were removed also because their inclusion in models led to serious multicollinearity (very high VIFs).

For compositional variables we removed Rhod, MountPine, MeadPastTree, Glacier, because those variables had zero values in most cases, thus were unlikely to contribute to the observed patterns, and GreenUrban because of high correlation with Urb (Spearman's Rho: GreenUrban = 0.68; $p < 0.001$). MixFor was removed because its inclusion in models led to serious multicollinearity (very high VIFs).

For configurational variables we left SHEI to high collinearity with SHDI (Spearman's Rho: SHEI = 0.87; $p < 0.001$). For variables full names see Table 1.

Table S1 Description of the bird traits used to calculate the functional dispersion indices and for the fourth-corner models. Conservation status was used only in the fourth-corner models

Type	Variable	Description	Unit	Source
Specialization	Specialism_index_overall	Index calculated by normalising the mean values of the five specialisation indices (diet specialism, foraging behaviour specialism, foraging substrate specialism, habitat specialism, nesting site specialism)	0-1 (index)	Morelli et al. 2019
Migration strategy	Sedentary	Species lives in the same area in both breeding and non-breeding season	0 = No; 1 = Yes	Storchová and Hořák 2018

Territoriality	Territoriality	Defence of a territory (defended area occupied exclusively by a single bird, pair, or larger social unit)		
Habitat usage during breeding season	Human_settlements	Species occupies LULC type in breeding area		
	Rocks			
	wetland			
	Open_area			
	Shrubland			
	Forest			
Nest type	Nest_typeOA	Open-arboreal, cup in bush, tree, on cliff ledge		
	Nest_typeCA	Closed-arboreal		
	Nest_typeGC	Ground close, nest in tussock very close to ground but not directly on ground, hidden in surrounded vegetation(s1) (G)		
	Nest_typeG	Ground, on ground directly		
	Nest_typeHP	Hole, in tree, bank, ground, crevice - occupying existing cavities		
	Nest_typeHA	Hole, in tree, bank, ground, crevice - active cavity builder		
Broods per year	Broods_per_year	Mean number of broods per breeding season (replaced broods are not included)	n	
Morphology	mean_body_mass	Mean body mass between sexes	cm	Dunning 2008; Storchová and Hořák 2018
Foraging substrate	ForStrat_water	Water layer	Prevalence of foraging behaviour - estimated % of use (sum of all = 100%).	Wilman et al. 2014
	ForStrat_ground	Herb and shrub layer		
	ForStrat_trees	Tree layer		
	ForStrat_aerial	Aerial layer		
Diet	Diet_Invertebrate	Invertebrate	Predominant diet among four categories. 0 = No; 1 = Yes.	
	Diet_VertFishScav	Vertebrate, fish, and carrion		
	Diet_PlantSeed	Plant and seeds		
	Diet_Omnivore	<= 50 in all three categories (Invertebrate, VertFishScav, PlantSeed)		
	CR	Critically Endangered		

Conservation status	EN	Endangered	0 = No; 1 = Yes	Ceresa and Kranebitter 2020
	VU	Vulnerable		
	NT	Near Threatened		
	LC	Least Concern		
	DD	Data were not sufficient to perform the assessment (Data Deficient)		

Table S2 List of the species censused during the bird survey.

Species	Code
**Species retained for analyses**	
Acanthis flammea	aca_fla
Accipiter gentilis	acc_gen
Accipiter nisus	acc_nis
Acrocephalus palustris	acr_pal
Acrocephalus scirpaceus	acr_sci
Aegithalos caudatus	aeg_cau
Alauda arvensis	ala_arv
Alcedo atthis	alc_att
Anas platyrhynchos	ana_pla
Anthus spinoletta	ant_spi
Anthus trivialis	ant_tri
Apus apus	apu_apu
Aquila chrysaetos	aqu_chr
Ardea cinerea	ard_cin
Aythya fuligula	ayt_ful
Buteo buteo	but_but
Carduelis carduelis	car_car
Certhia brachydactyla	cer_bra
Certhia familiaris	cer_fam
Cettia cetti	cet_cet
Chloris chloris	chl_chl
Cinclus cinclus	cin_cin
Circus aeruginosus	cir_aer
Columba livia	col_liv
Columba palumbus	col_pal
Corvus corone	cor_cor
Corvus corone cornix	cor_nix

Corvus corax	cor_rax
Coturnix coturnix	cot_cot
Crex crex	cre_cre
Cuculus canorus	cuc_can
Cyanistes caeruleus	cya_cae
Cygnus olor	cyg_olo
Delichon urbicum	del_urb
Dendrocopos major	den_maj
Dryocopus martius	dry_mar
Dryobates minor	dry_min
Emberiza cia	emb_cia
Emberiza citrinella	emb_cit
Emberiza hortulana	emb_hor
Erithacus rubecula	eri_rub
Falco subbuteo	fal_sub
Falco tinnunculus	fal_tin
Fringilla coelebs	fri_coe
Fulica atra	ful_atr
Gallinago gallinago	gar_gla
Hippolais polyglotta	hip_pol
Hirundo rustica	hir_rus
Ixobrychus minutus	ixo_min
Jynx torquilla	jyn_tor
Lagopus muta	lag_mut
Lanius collurio	lan_col
Larus michahellis	lar_mic
Linaria cannabina	lin_can
Lophophanes cristatus	lop_cri
Loxia curvirostra	lox_cur
Lullula arborea	lul_arb
Milvus migrans	mil_mig
Milvus milvus	mil_mil
Montifringilla nivalis	mon_niv
Monticola saxatilis	mon_sax
Motacilla alba	mot_alb
Motacilla cinerea	mot_cin
Muscicapa striata	mus_str
Nucifraga caryocatactes	nuc_car
Oenanthe oenanthe	oen_oen
Parus major	par_maj
Passer domesticus	pas_dom

Passer italiae	pas_ita
Passer montanus	pas_mon
Periparus ater	per_ate
Phoenicurus ochuros	pho_och
Phoenicurus phoenicurus	pho_pho
Phylloscopus bonelli	phy_bon
Phylloscopus collybita	phy_col
Phylloscopus trochilus	phy_tro
Picus canus	pic_can
Pica pica	pic_pic
Picoides tridactylus	pic_tri
Picus viridis	pic_vir
Podiceps cristatus	pod_cri
Poecile montanus	poe_mon
Poecile palustris	poe_pal
Prunella collaris	pru_col
Prunella modularis	pru_mod
Ptyonoprogne rupestris	pty_rup
Pyrrhocorax graculus	pyr_gra
Pyrrhula pyrrhula	pyr_pyr
Regulus ignicapilla	reg_ign
Regulus regulus	reg_reg
Saxicola rubetra	sax_rub
Serinus serinus	ser_ser
Sitta europaea	sit_eur
Spinus spinus	spi_spi
Streptopelia decaocto	str_dec
Strix aluco	stri_alu
Sturnus vulgaris	stu_vul
Sylvia atricapilla	syl_atr
Sylvia communis	syl_com
Sylvia curruca	syl_cur
Tachymarptis melba	tac_mel
Tachybaptus ruficollis	tac_ruf
Tichodroma muraria	tic_mur
Troglodytes troglodytes	tro_tro
Turdus merula	tur_mer
Turdus philomelos	tur_phi
Turdus pilaris	tur_pil
Turdus torquatus	tur_tor
Turdus viscivorus	tur_vis

Upupa epops	upu_epo
**migrant / not breeding species**	
Actitis hypoleucos	act_hyp
Casmerodius albus	cas_alb
Ficedula hypoleuca	fic_hyp
Locustella naevia	loc_nae
Ardea purpurea	ard_pur

Table S3 Most supported models on the effect of environmental predictors on breeding species richness (Sric), Shannon diversity (Shan), and functional dispersion (Fids), at both 100 and 400 m spatial scales. Models are ranked according to Akaike's information criterion corrected for small sample size (AICc) and only models within an interval of ΔAICc < 2 are shown. The differences in AICc from the best supported model (ΔAICc), Akaike's weights (wi), and -2 log-likelihood values (logLik) are also given. Negative (-) or positive (+) relationships between predictors and diversity indices are shown. Models including uninformative parameters are left out, see methods for details, and for variable full names see Table 1. Tables S3 can be found in a separate excel file named "*Table_S3_SUPPInfo.xlsx*"

Table S4 Averaged models standardised parameter (β; based on models with ΔAICc < 2) and standard error (SE) for the most supported models for species richness, Shannon diversity, and functional diversity at both spatial scales (100 and 400 m). See methods for details, and for variable full names see Table 1

Scale	Dependent variable	Model type	Parameter	β	SE	Z	P
100 m	Species richness	topo-cllimatical	(Intercept)	2.39	0.02	101.53	<0.001
			AnnPrec	-0.18	0.03	-7.15	<0.001
		compositional	**Parameter**	**β**	**SE**	**Z**	**P**
			(Intercept)	2.37	0.02	96.90	<0.001
			AlpGrass	-0.14	0.03	4.62	<0.001
			RocScr	-0.21	0.04	5.70	<0.001
			PermCult	-0.06	0.03	2.41	0.02
			LakRiv	0.04	0.02	2.14	0.03

		Parameter	β	SE	Z	P
		Wet	0.05	0.02	2.45	0.01
		Roads	0.03	0.02	0.93	0.35
	**configurational**	**Parameter**	**β**	**SE**	**Z**	**P**
		(Intercept)	2.40	0.02	102.58	<0.001
		PR	0.13	0.02	5.87	<0.001
	**synthetic (averaged)**	**Parameter**	**β**	**SE**	**Z**	**P**
		(Intercept)	2.37	0.02	96.63	<0.001
		AnnPrec	-0.02	0.03	0.61	0.54
		AlpGrass	-0.13	0.03	4.03	<0.001
		RocScr	-0.20	0.04	4.95	<0.001
		PermCult	-0.06	0.03	2.19	0.03
		LakRiv	0.01	0.02	0.58	0.56
		Wet	0.05	0.02	2.50	0.01
		PR	0.07	0.02	3.01	0.003
**Shannon diversity**	**topo-cllimatical**	**Parameter**	**β**	**SE**	**Z**	**P**
		(Intercept)	2.09	0.03	61.75	<0.001
		AnnPrec	-0.21	0.03	-6.08	<0.001
	**compositional**	**Parameter**	**β**	**SE**	**Z**	**P**
		(Intercept)	2.09	0.03	70.69	<0.001
		AlpGrass	-0.17	0.03	5.25	<0.001
		RocScr	-0.22	0.03	6.78	<0.001
		AnnCult	-0.08	0.03	2.46	0.01
		PermCult	-0.09	0.03	2.80	0.005
		Wet	0.02	0.03	0.63	0.53
		Roads	0.04	0.04	1.13	0.26
		Urb	-0.12	0.03	3.83	<0.001
	**configurational**	**Parameter**	**β**	**SE**	**Z**	**P**
		(Intercept)	2.09	0.04	59.65	<0.001
		PR	0.17	0.04	4.84	<0.001
	**synthetic (averaged)**	**Parameter**	**β**	**SE**	**Z**	**P**
		(Intercept)	2.09	0.03	73.31	<0.001
		AnnPrec	-0.13	0.04	3.09	0.002
		AlpGrass	-0.13	0.03	3.78	<0.001
		RocScr	-0.17	0.03	4.99	<0.001
		AnnCult	-0.10	0.03	3.19	0.001
		PermCult	-0.12	0.03	3.19	<0.001
		Roads	0.02	0.03	0.58	0.56
		Urb	-0.13	0.03	4.19	<0.001
		PR	0.05	0.04	1.12	0.26

Response	Model	Parameter	β	SE	Z	P
**Functional dispersion**	**topo-climatical**	(Intercept)	0.17	0.003	52.11	<0.001
		SolarRad	0.00	0.004	1.09	0.28
		AnnPrec	-0.01	0.003	3.45	<0.001
	**compositional**	(Intercept)	0.17	0.003	65.10	<0.001
		RocScr	-0.004	0.004	0.85	0.40
		AlpGrass	0.005	0.005	1.10	0.27
		AlpShr	0.002	0.003	0.64	0.52
		Meadow	0.02	0.005	3.50	<0.001
		AnnCult	0.01	0.004	2.53	0.01
		PermCult	-0.003	0.004	0.66	0.51
		HedgShru	-0.002	0.003	0.72	0.47
		ConFor	-0.003	0.004	0.69	0.49
		DecFor	0.001	0.003	0.52	0.60
		LakRiv	0.01	0.003	1.91	0.06
		Wet	0.008	0.003	2.50	0.01
		Roads	0.005	0.004	1.35	0.18
		Urb	0.02	0.004	5.26	<0.001
	**configurational**	(Intercept)	0.17	0.003	51.83	<0.001
		AREAMN	-0.01	0.003	-3.11	0.002
	**synthetic (averaged)**	(Intercept)	0.17	0.003	65.44	<0.001
		SolarRad	-0.01	0.003	1.78	0.07
		AnnPrec	-0.01	0.003	1.77	0.08
		AlpGrass	0.01	0.004	2.09	0.04
		RocScr	-0.002	0.003	0.62	0.54
		AlpShr	0.003	0.003	1.00	0.32
		Meadow	0.02	0.004	5.12	<0.001
		AnnCult	0.01	0.003	3.26	0.001
		PermCult	-0.001	0.002	0.24	0.81
		HedgShru	-0.003	0.003	0.94	0.35
		DecFor	0.0006	0.002	0.33	0.74
		ConFor	-0.0005	0.002	0.25	0.81
		LakRiv	0.007	0.003	2.42	0.02
		Wet	0.008	0.003	3.00	0.003
		Roads	0.004	0.004	1.01	0.31
		Urb	0.02	0.003	7.10	<0.001
		AREAMN	-0.002	0.003	0.59	0.56

Scale	Response	Model	Parameter	β	SE	Z	P
400 m	Species richness	topo-cllimatical	(Intercept)	2.39	0.02	101.53	<0.001
			AnnPrec	-0.18	0.03	-7.15	<0.001
		compositional	(Intercept)	2.36	0.02	95.43	<0.001
			RocScr	-0.18	0.04	4.59	<0.001
			AlpGrass	-0.15	0.03	4.21	<0.001
			Meadow	0.08	0.03	3.16	0.002
			Pasture	0.01	0.02	0.52	0.600
			AnnCult	-0.03	0.03	0.82	0.415
			DecFor	0.06	0.02	2.61	0.009
			LakRiv	0.08	0.02	3.81	<0.001
		configurational	(Intercept)	2.38	0.02	100.27	<0.001
			PR	0.22	0.04	5.77	<0.001
			AREAMN	-0.09	0.04	-2.54	0.011
			SHDI	-0.08	0.04	-2.29	0.022
		synthetic (averaged)	(Intercept)	2.35	0.02	94.91	<0.001
			AnnPrec	0.05	0.04	1.51	0.130
			RocScr	-0.17	0.04	4.18	<0.001
			AlpGrass	-0.16	0.04	4.38	<0.001
			Meadow	0.02	0.03	0.64	0.52
			Pasture	0.01	0.02	0.50	0.617
			AnnCult	-0.03	0.03	0.91	0.364
			LakRiv	0.07	0.02	3.40	<0.001
			PR	0.12	0.03	4.48	<0.001
	Shannon diversity	topo-cllimatical	(Intercept)	2.09	0.03	61.75	<0.001
			AnnPrec	-0.21	0.03	-6.08	<0.001
		compositional	(Intercept)	2.09	0.03	75.51	<0.001
			AlpGrass	-0.19	0.04	5.25	<0.001
			RocScr	-0.19	0.03	5.68	<0.001
			Meadow	0.04	0.04	0.85	0.40
			AnnCult	-0.08	0.03	2.54	0.01
			PermCult	-0.05	0.04	1.09	0.28
			DecFor	0.02	0.03	0.72	0.47
			LakRiv	0.07	0.03	2.52	0.01
			Roads	0.01	0.03	0.46	0.64
			Urb	-0.11	0.03	3.36	<0.001

		Parameter	β	SE	Z	P
	configurational	(Intercept)	2.09	0.03	64.63	<0.001
		PR	0.27	0.05	5.25	<0.001
		AREAMN	-0.08	0.04	-2.08	0.04
		SHDI	-0.12	0.05	-2.47	0.01
	synthetic (averaged)	**Parameter**	**β**	**SE**	**Z**	**P**
		(Intercept)	2.09	0.03	78.30	<0.001
		AlpGrass	-0.19	0.03	5.92	<0.001
		RocScr	-0.16	0.03	4.96	<0.001
		AnnCult	-0.08	0.03	2.57	0.01
		PermCult	-0.05	0.03	1.37	0.17
		LakRiv	0.07	0.03	2.37	0.02
		Urb	-0.10	0.03	3.63	<0.001
		PR	0.10	0.04	2.60	0.01
		AREAMN	-0.03	0.04	0.74	0.46
Functional dispersion	topo-cllimatical	**Parameter**	**β**	**SE**	**Z**	**P**
		(Intercept)	0.17	0.003	52.11	<0.001
		SolarRad	0.004	0.004	1.09	0.28
		AnnPrec	-0.01	0.003	3.45	<0.001
	compositional	**Parameter**	**β**	**SE**	**Z**	**P**
		(Intercept)	0.17	0.003	64.14	<0.001
		RocScr	-0.003	0.003	1.03	0.30
		AlpShr	0.004	0.003	1.42	0.15
		Meadow	0.02	0.003	6.14	<0.001
		AnnCult	0.003	0.003	0.84	0.40
		RipForest	-0.005	0.003	1.59	0.11
		HedgShru	-0.0007	0.002	0.33	0.74
		LakRiv	0.01	0.003	3.77	<0.001
		Urb	0.02	0.003	6.44	<0.001
	configurational	**Parameter**	**β**	**SE**	**Z**	**P**
		(Intercept)	0.17	0.003	54.40	<0.001
		AREAMN	-0.02	0.003	-5.25	<0.001
	synthetic (averaged)	**Parameter**	**β**	**SE**	**Z**	**P**
		Intercept	0.17	0.003	65.23	<0.001
		AlpShr	0.003	0.003	0.90	0.37
		Meadow	0.02	0.003	5.67	<0.001
		AnnCult	0.002	0.003	0.66	0.51
		RipForest	-0.004	0.003	1.11	0.27
		HedgShru	-0.003	0.004	0.87	0.38
		LakRiv	0.01	0.003	3.61	<0.001
		Urb	0.02	0.003	6.33	<0.001
		AREAMN	-0.009	0.003	2.94	0.003

Table S5 Environmental variables sources. For more details see Table 1

Type	Variable	Name	Sources
Topo-climatical	SolarRad	Potential solar radiation	Digital Elevation Model (DEM 2020)
	TMAMme	Mean spring temperature	Tscholl et al. 2021
	TANNUALme	Mean annual temperature	
	Elev	Elevation	Digital Elevation Model (DEM 2020)
	Slope	Slope	
	AnnPrec	Mean annual precipitation sum	Rubel et al. 2017
Compositional	Glacier	Glaciers	OpenStreetMap data (Geofabrik 2020)
	Urb	Urban areas	
	GreenUrban	Green urban	
	AlpGrass	Alpine grasslands and summer pastures	LAFIS (LAFIS 2020)
	AlpShr	Highly structured grasslands	
	HedgShru	Hedges and/or shrubs	
	Meadow	Hay meadows	
	Pasture	Pastures	
	MeadPastTree	Meadows and pastures with trees and/or bushes	

	AnnCult	Annual crops	
	PermCult	Permanent crops	
	RocScr	Rock/ screen slopes	GeoCatalogo (GeoCatalogo 2020)
	DecFor	Deciduous forests	GeoCatalogo (GeoCatalogo 2020)
	ConFor	Coniferous forests	GeoCatalogo (GeoCatalogo 2020)
	RipFor	Riparian forests	GeoCatalogo (GeoCatalogo 2020)
	MixFor	Mixed forests	GeoCatalogo (GeoCatalogo 2020)
	MountPine	Mountain pines	GeoCatalogo (GeoCatalogo 2020)
	Rhod	Rhododendrons	GeoCatalogo (GeoCatalogo 2020)
	Wet	Wetlands	GeoCatalogo (GeoCatalogo 2020)
	LakRiv	Lakes and rivers	OpenStreetMap data (Geofabrik 2020)
	Roads	Roads, tracks and rail	OpenStreetMap data (Geofabrik 2020)
Configurational	ED	Edge density	FRAGSTATS (McGarigal 2015)
Configurational	AREA_MN	Mean patch area	FRAGSTATS (McGarigal 2015)
Configurational	PR	Patch richness	FRAGSTATS (McGarigal 2015)
Configurational	SHDI	Shannon diversity index	FRAGSTATS (McGarigal 2015)
Configurational	SHEI	Shannon evenness index	FRAGSTATS (McGarigal 2015)

Fig. S1 Graphical representation of the effect of environmental drivers on species richness as predicted by averaged synthetic model at 100-m spatial scale. Other predictors included in the models are kept constant at their mean value. 95% CI of the mean are shown in grey

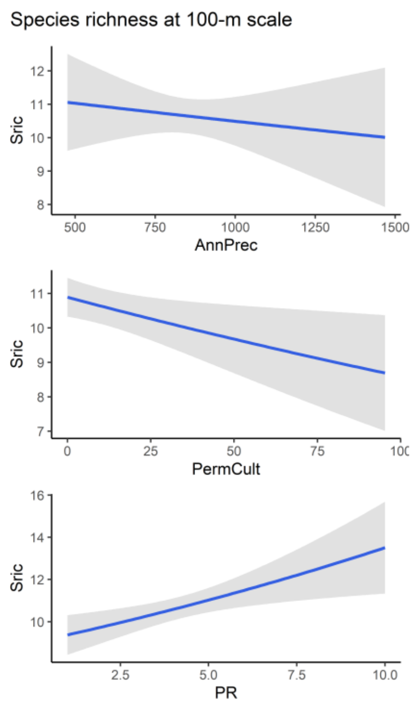

Fig. S2 Graphical representation of the effect of environmental drivers on species richness as predicted by averaged synthetic model at 400-m spatial scale. Other predictors included in the models are kept constant at their mean value. 95% CI of the mean are shown in grey

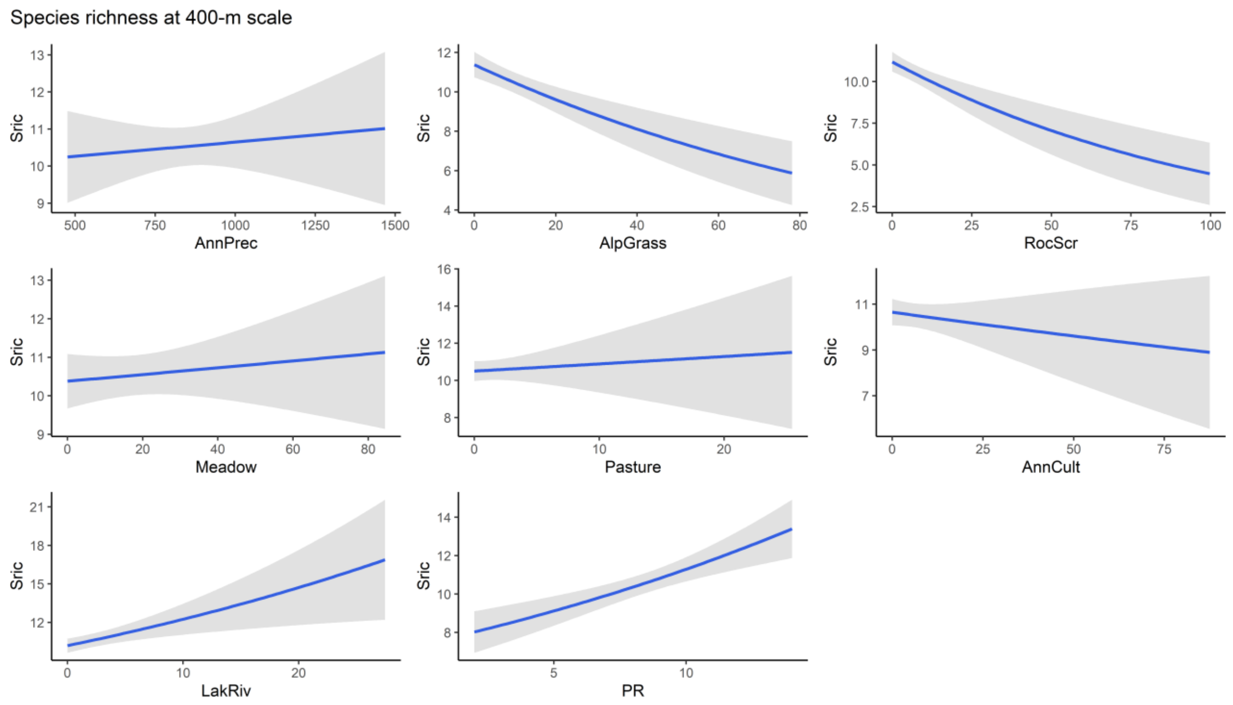

Fig. S3 Graphical representation of the effect of environmental drivers on Shannon diversity as predicted by averaged synthetic model at 100-m spatial scale. Other predictors included in the models are kept constant at their mean value. 95% CI of the mean are shown in grey

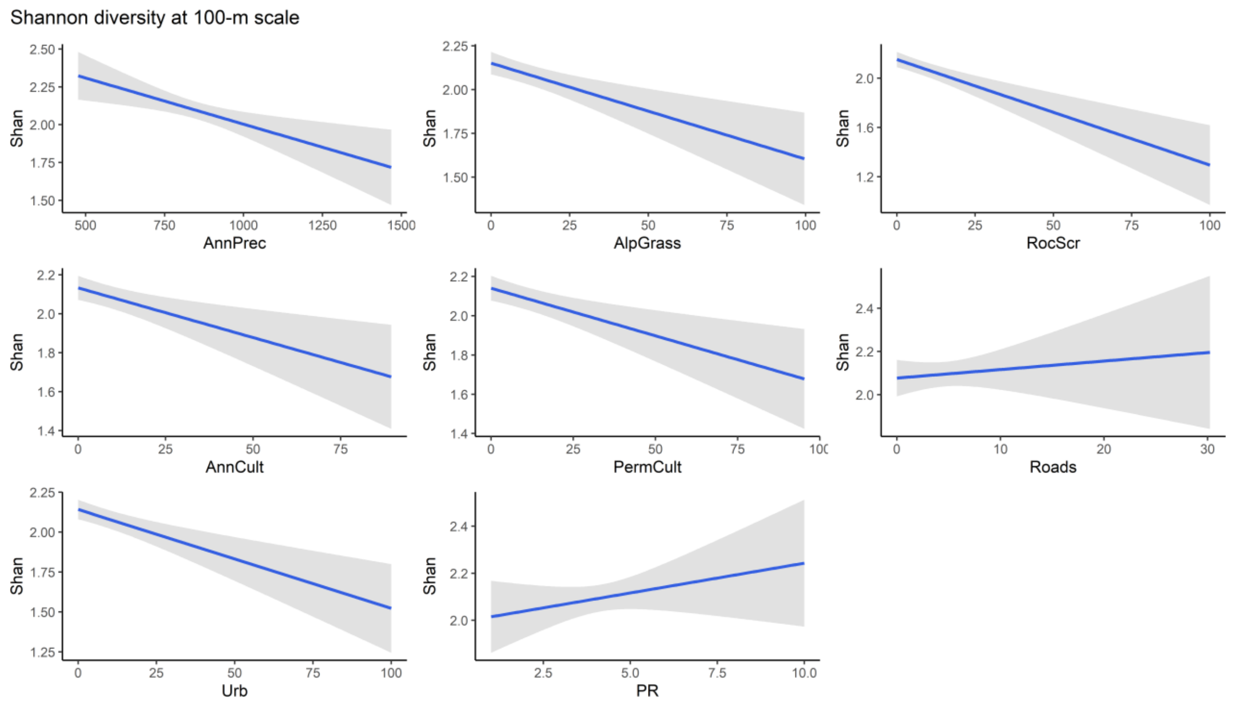

Fig. S4 Graphical representation of the effect of environmental drivers on Shannon diversity as predicted by averaged synthetic model at 400-m spatial scale. Other predictors included in the models are kept constant at their mean value. 95% CI of the mean are shown in grey

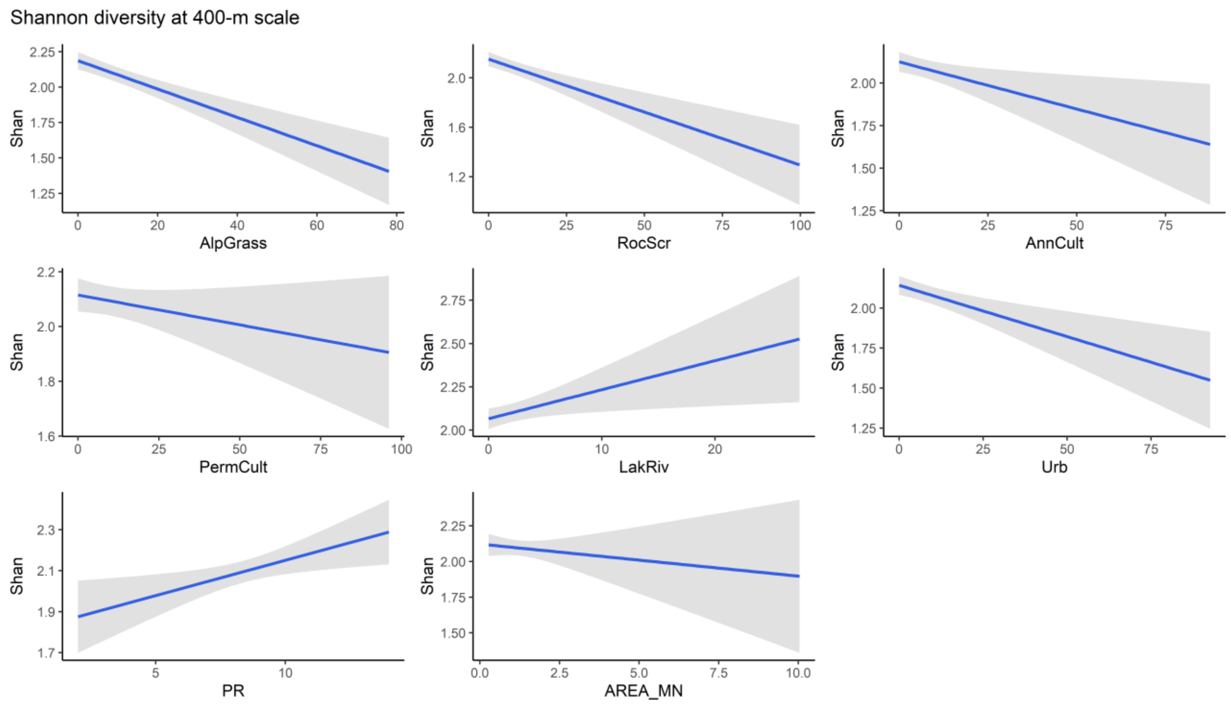

Fig. S5 Graphical representation of the effect of environmental drivers on functional dispersion as predicted by averaged synthetic model at 100-m spatial scale. Other predictors included in the models are kept constant at their mean value. 95% CI of the mean are shown in grey

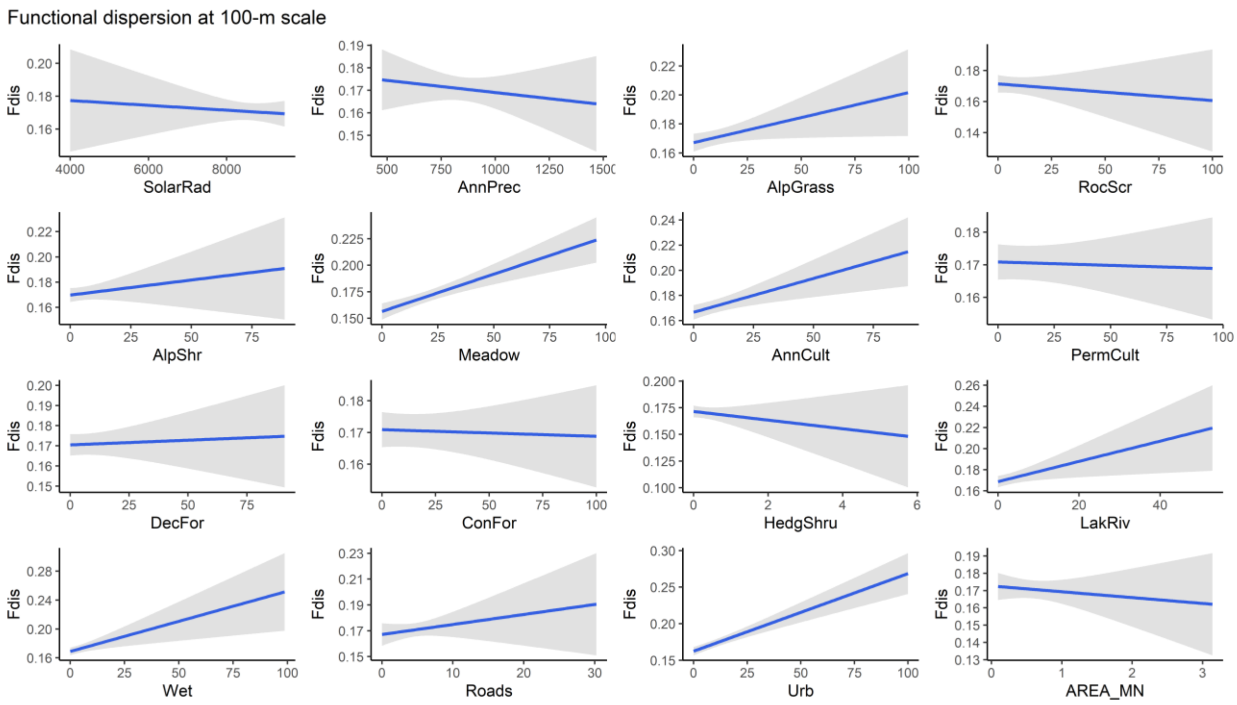

Fig. S6 Graphical representation of the effect of environmental drivers on functional dispersion as predicted by averaged synthetic model at 400-m spatial scale. Other predictors included in the models are kept constant at their mean value. 95% CI of the mean are shown in grey

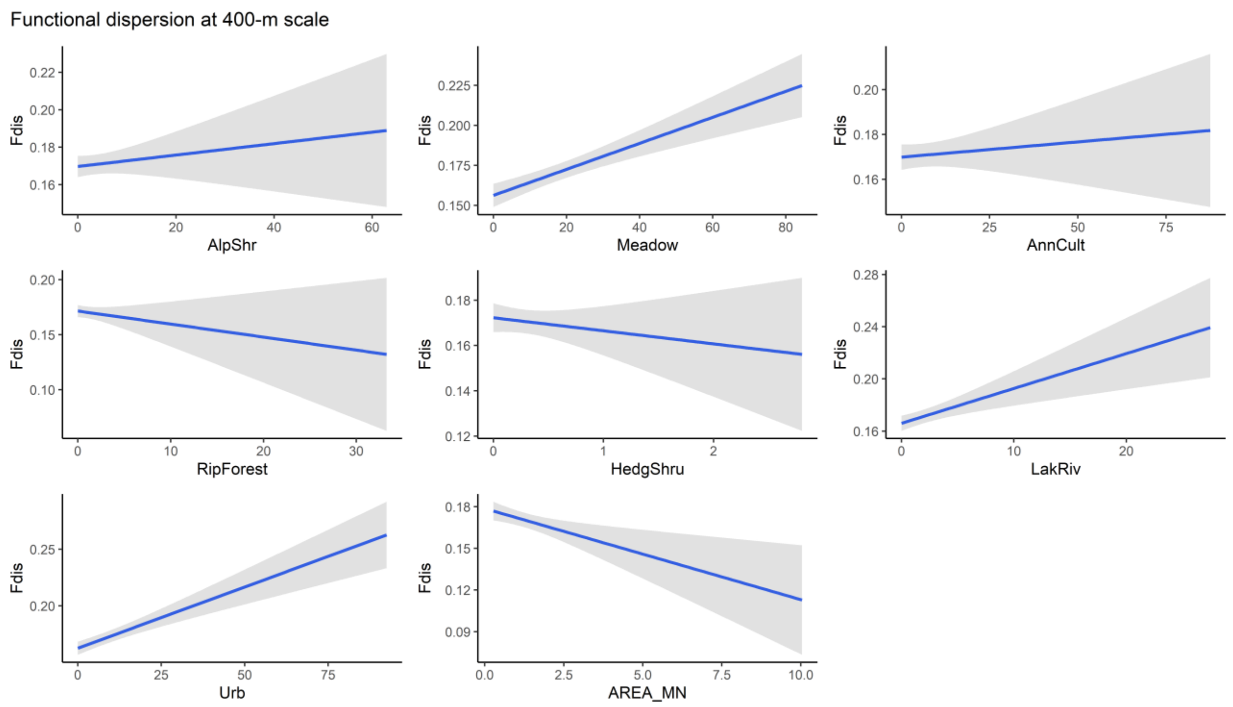

Fig. S7 Accumulation curves based on abundance of individuals recorded during the surveys (number of sites = 168). We used Hill number N1 to down weights rare species. Here we show three types of plots: sample-size-based rarefaction/extrapolation curve (A); sample completeness curve (B); coverage-based rarefaction/extrapolation curve (C). Shaded areas: 95% confidence limits of the curves

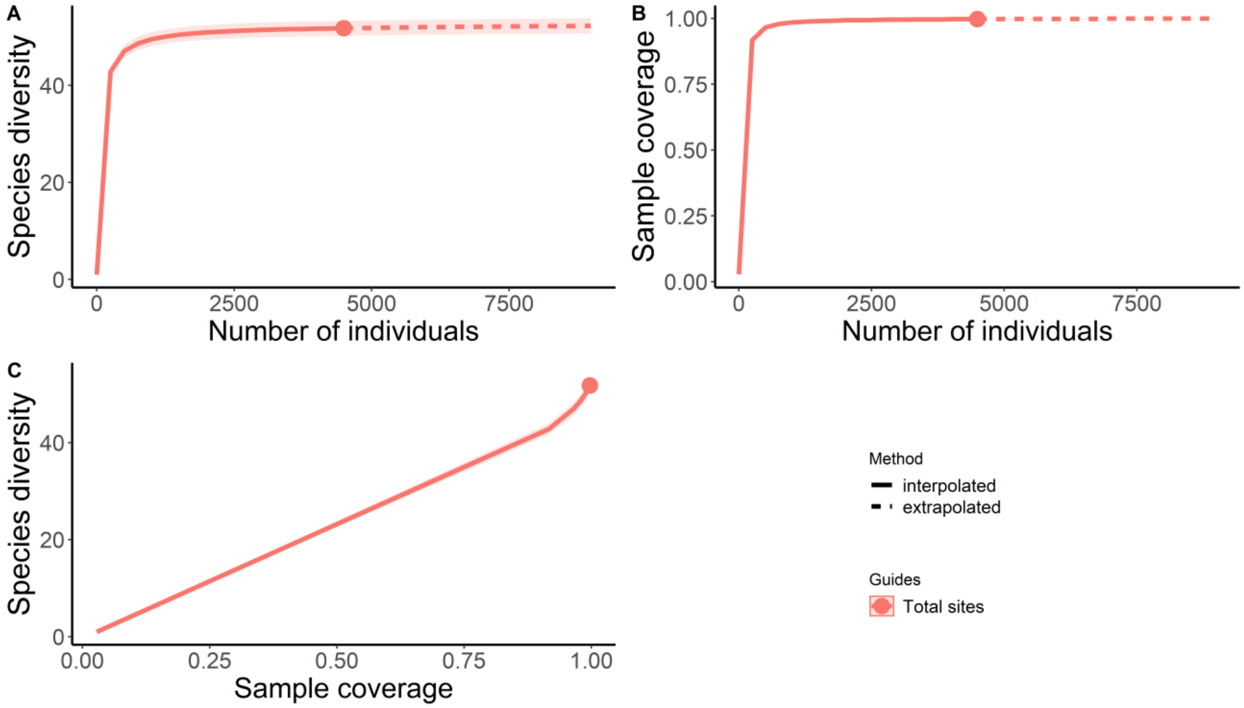

Fig. S8 Accumulation curves based on abundance of individuals recorded during the surveys of the alpine/subalpine sites (number of sites =40). We used Hill number N1 to down weights rare species. Here we show three types of plots: sample-size-based rarefaction/extrapolation curve (A); sample completeness curve (B); coverage-based rarefaction/extrapolation curve (C). Shaded areas: 95% confidence limits of the curves

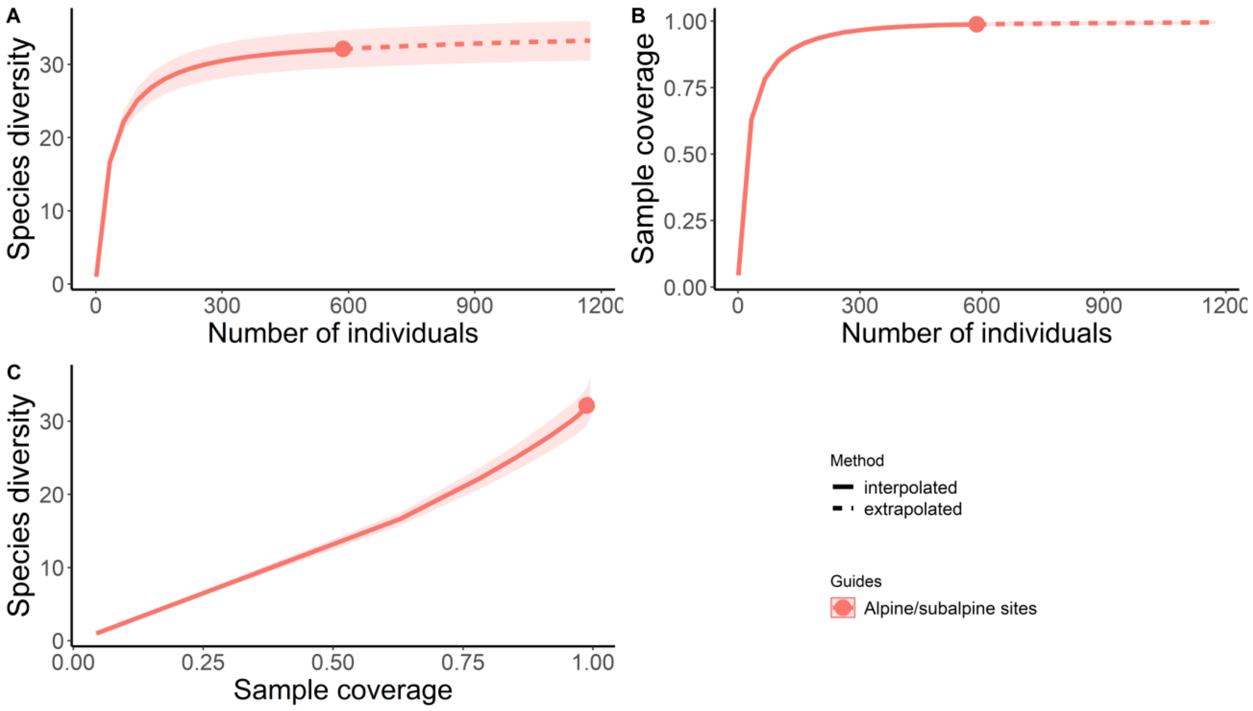

References

Ceresa, F., Kranebitter Petra (2020) Lista Rossa 2020 degli uccelli nidificanti in Alto Adige. https://doi.org/10.5281/ZENODO.4245030

DEM (2020) Digital Elevation Model. http://geoservices.buergernetz.bz.it/geoserver/p_bz-elevation/ows?SERVICE=WMS. Accessed 5 Jan 2021

Dunning JB (ed) (2008) CRC handbook of avian body masses, 2nd ed. CRC Press, Boca Raton

GeoCatalogo (2020) Rete Civica dell’Alto Adige. http://geocatalogo.retecivica.bz.it/geokatalog/#!

Geofabrik (2020) OpenStreetMap. http://www.geofabrik.de/. Accessed 5 Jan 2020

LAFIS (2020) Amt für landwirtschaftliche Informationssysteme. http://www.provinz.bz.it/de/kontakt.asp?orga_orgaid=948. Accessed 5 Jan 2020

McGarigal K (2015) FRAGSTATS Help, Version 4.2. University of Massachusetts Amherst

Morelli F, Benedetti Y, Møller AP, Fuller RA (2019) Measuring avian specialization. Ecol Evol 9:8378–8386. https://doi.org/10.1002/ece3.5419

Rubel F, Brugger K, Haslinger K, Auer I (2017) The climate of the European Alps: Shift of very high resolution Köppen-Geiger climate zones 1800-2100. Meteorol Z 26:115–125. https://doi.org/10.1127/metz/2016/0816

Storchová L, Hořák D (2018) Life-history characteristics of European birds. Glob Ecol Biogeogr 27:400–406. https://doi.org/10.1111/geb.12709

Tscholl S, Tasser E, Ulrike T, Lukas EV (2021) Coupling solar radiation and cloud cover data for enhanced temperature predictions over topographically complex mountain terrain. Int J Climatol joc.7497. https://doi.org/10.1002/joc.7497

Wilman H, Belmaker J, Simpson J, et al (2014) EltonTraits 1.0: Species-level foraging attributes of the world’s birds and mammals: *Ecological Archives* E095-178. Ecology 95:2027–2027. https://doi.org/10.1890/13-1917.1
